# Relative and absolute reliability of medial knee joint space width measurement using ultrasound imaging during valgus stress testing

**DOI:** 10.1371/journal.pone.0358761

**Published:** 2026-09-18

**Authors:** Ryoga Hayashi, Hayato Kawaji, Satoru Kojima

**Affiliations:** 1 Nantaru Orthopedic Clinic, Otaru, Hokkaido, Japan; 2 Soseigawadori Orthopedic Clinic, Sapporo, Hokkaido, Japan; 3 School of Rehabilitation Sciences, Health Sciences University of Hokkaido, Kanazawa, Tobetsu-cho, Japan; Cairo University Faculty of Physical Therapy, EGYPT

## Abstract

This study aimed to evaluate both the relative and absolute reliability of ultrasound-based measurements of changes in medial knee joint space width under valgus stress in healthy individuals. Seventeen healthy adult males (34 knees) underwent ultrasound assessments under unloaded and valgus-loaded (10 Nm) conditions. The medial knee joint space width was measured using a standardized ultrasound protocol, and changes were calculated as the difference between loaded and unloaded conditions. Intra- and inter-rater reliability between two physical therapists with different experience levels were evaluated using intraclass correlation coefficients, and absolute reliability was assessed via Bland-Altman analysis, standard error of measurement, and minimal detectable change at the 95% confidence level. A significant increase in medial knee joint space width (ranging from 1.33 to 1.41 mm) was observed under valgus stress compared to the unloaded condition (*p* < 0.001). The intraclass correlation coefficient for intra-rater reliability across two separate measurement days was 0.88 (95% confidence interval: 0.77–0.94), whereas that for inter-rater reliability was 0.84 (95% confidence interval: 0.70–0.92). Bland-Altman analysis revealed only random measurement error, with minimal detectable change at the 95% confidence level values below 1.0 mm (0.51 mm intra-rater; 0.65 mm inter-rater). Ultrasound imaging could provide sufficient reliability to quantify changes in medial knee joint space width during valgus stress testing in healthy individuals. These values suggest that changes exceeding these thresholds may reflect changes beyond measurement error. However, further studies with larger sample sizes and involving patients with ligamentous injuries are needed to confirm the clinical applicability of this method.

## Introduction

Injuries to the medial structures of the knee, particularly the medial collateral ligament (MCL), are common and may result in medial knee instability [1–5]. The valgus stress test is widely used to evaluate the integrity of these structures [3–6]. However, the test relies largely on the examiner’s subjective assessment of the medial joint opening, which may reduce its objectivity and reproducibility [7]. To overcome this limitation, quantitative assessment methods have been developed. Although stress radiography is considered a reference method for evaluating medial knee laxity [8,9], its clinical use is limited by radiation exposure and equipment requirements. Ultrasound imaging has emerged as an attractive alternative because it is non-invasive, radiation-free, portable, and capable of providing real-time dynamic assessment of medial knee structures [10–13].

Previous studies have demonstrated that ultrasound measurements of changes in medial knee joint space width under valgus stress correlate well with stress radiography [10,12] and exhibit high intra- and inter-rater reliability [10,11]. These findings suggest that ultrasound may be useful for quantifying medial knee laxity. However, previous studies have primarily focused on relative reliability, which reflects measurement consistency but does not indicate the type or magnitude of measurement error. For clinical decision-making, absolute reliability is equally important because it enables determination of whether an observed change exceeds measurement error and likely represents a change beyond measurement error. Parameters such as the standard error of measurement (SEM) and minimal detectable change at the 95% confidence level (MDC_95_) provide this information. Although absolute reliability of ultrasound measurements has been reported for several knee laxity-related assessments [14,15], no study has investigated the absolute reliability of ultrasound-based measurements of changes in medial knee joint space width under valgus stress. Consequently, the threshold for distinguishing changes beyond measurement error in medial joint space width remains unknown.

Therefore, the purpose of this study was to evaluate both the relative and absolute reliability of ultrasound-based measurements of changes in medial knee joint space width under valgus stress in healthy individuals. Establishing SEM and MDC_95_ values would provide useful thresholds for distinguishing changes beyond measurement error and may facilitate the clinical application of ultrasound-based assessment of medial knee laxity. We hypothesized that: (a) the measurement of changes in medial knee joint space width under valgus stress would demonstrate high relative reliability both within and between examiners, and (b) when assessing absolute reliability, the errors would be primarily random, with the magnitude of the errors being less than 1.0 mm, both within and between examiners.

## Materials and methods

### Participants

The sample size was originally determined to detect significant differences in medial knee joint space width between unloaded and loaded conditions. Sample size estimation was performed using G*power software (Version 3.1.9.7; Heinrich Heine University, Düsseldorf, Germany) based on a paired *t* test with a two-tailed analysis, assuming an effect size of 0.50, an alpha level of 0.05, and statistical power (1 – *β*) of 0.80. The results indicated that a sample size of 34 was required.

Given the influence of hormonal fluctuations on joint laxity in females [16–18], only male participants were included in this study to eliminate potential confounding effects. Participants were recruited through convenience sampling from the students at our university. Exclusion criteria included a history of knee surgery or trauma, subjective knee pain, severe leg malalignment, inflammatory diseases affecting the knee joint, and BMI ≥ 30. Consequently, 17 healthy adult males (34 knees) were enrolled in this study. Participants were prospectively recruited between May 24, 2022, and September 6, 2022. Written informed consent was obtained from all participants. This study was approved by the Ethics Committee of Health Sciences University of Hokkaido (Approval No. 22R172175).

### Procedures

The medial knee joint space width was measured using a diagnostic ultrasound system (Noblus; Hitachi Medical Corp., Tokyo, Japan) equipped with an 8–15 MHz linear transducer. Two licensed physical therapists performed the measurements. Examiner 1 was in their second year of clinical practice, and Examiner 2 had over 10 years of clinical experience. Two examiners (Examiners 1 and 2) underwent prior training until they achieved intra-rater reliability (ICC ≥ 0.80), as demonstrated in previous studies [10,11]. Subsequently, the formal ultrasound measurements were performed. A third examiner (Examiner 3), a licensed physical therapist with over 10 years of experience in musculoskeletal rehabilitation, applied valgus stress during the measurement.

**Fig 1:**
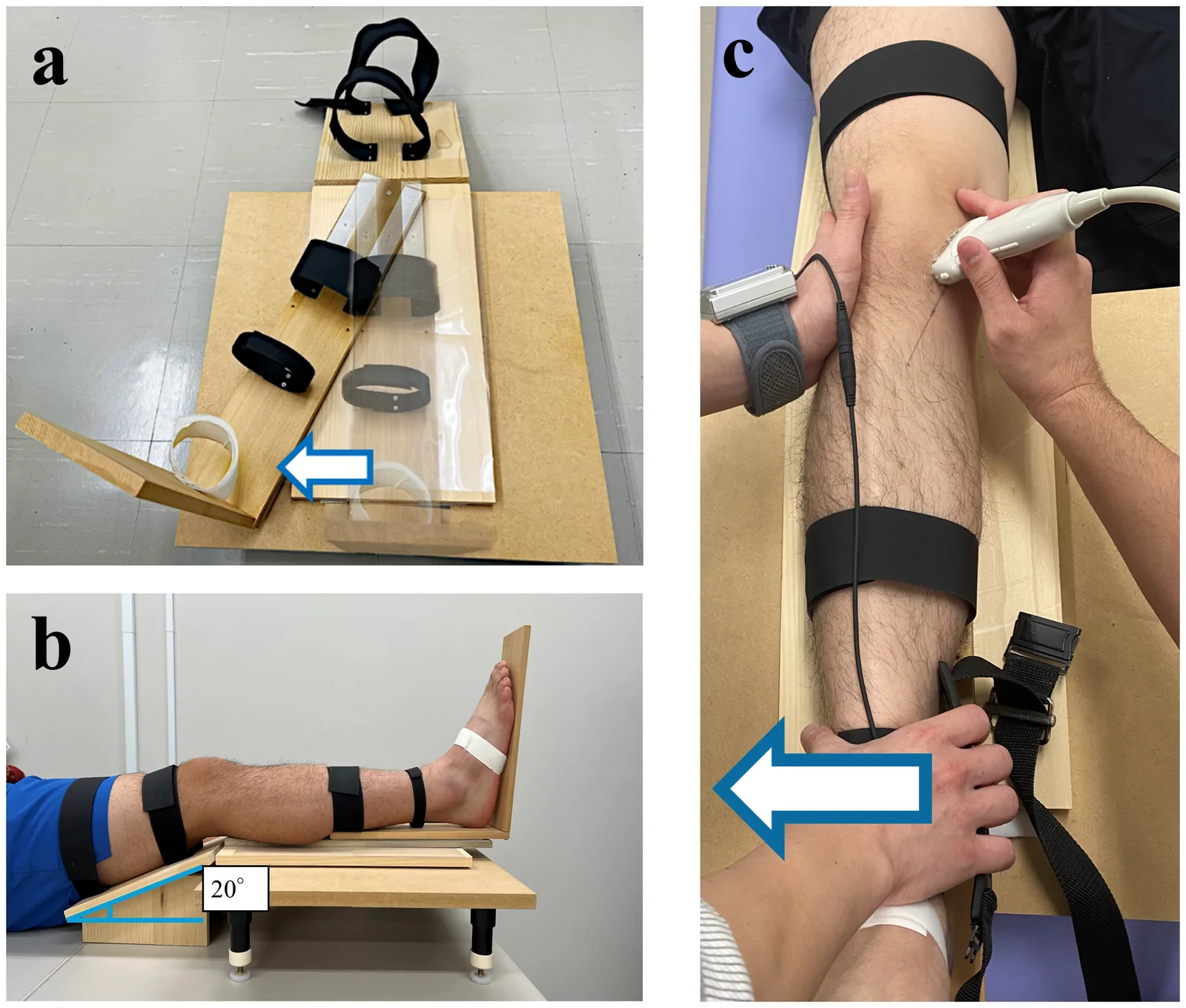
(a) A custom-made fixation device. **(b)** Knee fixed at 20° of flexion and ankle positioned in neutral dorsiflexion using the device. **(c)** Measurement of medial knee joint space width using ultrasound imaging.

Participants were positioned supine with the knees fixed at 20° flexion and the ankles in neutral dorsiflexion using a custom-made fixation device (Figs 1a and 1b). Examiners identified the MCL attachment sites on the femur and tibia via ultrasound imaging. These sites were marked with a pen and connected with a straight line to guide probe placement. The ultrasound probe was aligned along this line to visualize the long axis of the MCL. The probe was then positioned parallel to the anterior edge of the MCL and adjusted to achieve clear visualization of the bone boundary. Images were acquired repeatedly until three successful trials were obtained.

Ultrasound images were acquired under both unloaded and loaded conditions. For loaded condition measurements, a valgus load of 10 Nm was applied to the knee [8,10,19]. Examiner 3 stabilized the lateral side of the knee with one hand and applied valgus force to the medial malleolus using a handheld dynamometer (µTas F1; Anima Corp., Tokyo, Japan) with the other hand (Fig 1c). The valgus force was calculated by dividing 10 Nm by the length of the lower leg for each subject. The applied force was continuously monitored to ensure consistency throughout the procedure.

Ultrasound images were captured on two separate occasions: Day 1 and Day 2 (one week later). Imaging on Day 1 was performed by Examiner 1, while imaging on Day 2 was conducted by both Examiner 1 and 2 to assess intra- and inter-rater reliability. For the reliability assessments across the two separate measurement days, the skin markings were removed and re-identified to ensure independence. However, for same measurement day inter-rater assessments, the markings were retained.

**Fig 2:**
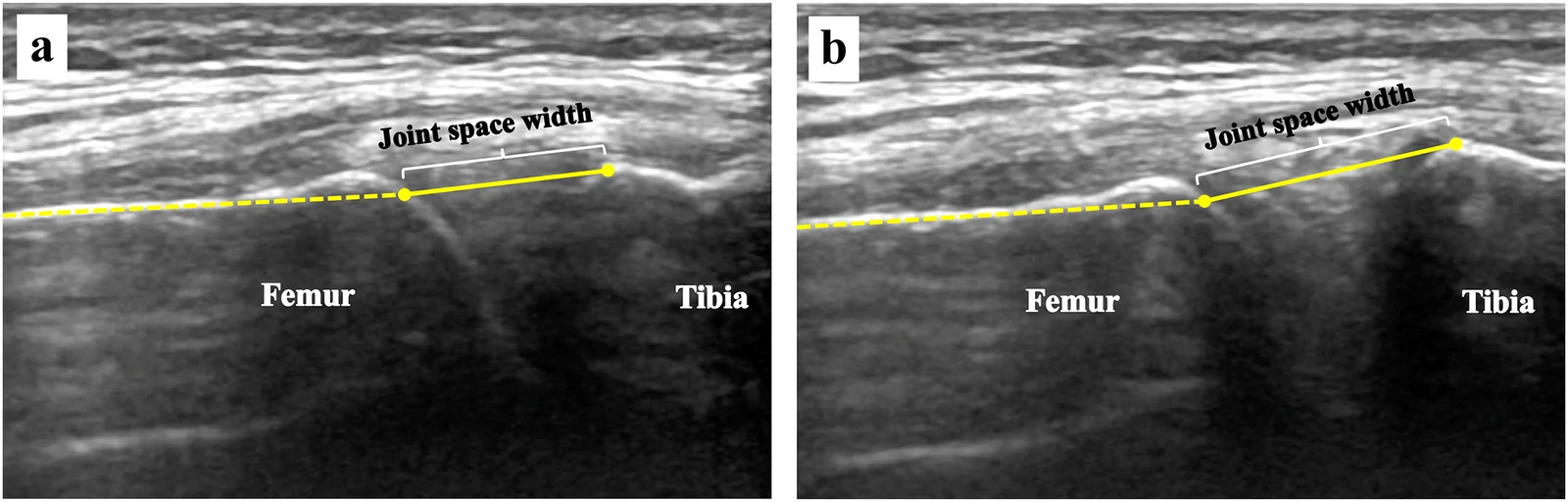
Ultrasound images of the medial knee joint space width. **(a)** Unloaded condition. **(b)** Loaded condition. The joint space width was measured as the distance (solid yellow line) between the distal femur end, defined by the extension of the medial femoral margin (dashed yellow line), and the most proximal medial bone surface of the tibial plateau.

Ultrasound images were imported into a PC and analyzed using ImageJ software (Version 1.53; National Institutes of Health, USA). The medial knee joint space width was defined as the distance between the distal end of the medial femoral edge and the most proximal medial surface of the tibia plateau along the extension of the medial femoral edge [20] (Fig 2). Image scale calibration was performed, and measurements were quantified using the distance measurement tool in ImageJ. Changes in the medial knee joint space width were calculated by subtracting the values under unloaded conditions from those under loaded conditions. All image analyses were performed by Examiner 1. To minimize bias, these analyses were conducted in a blinded manner; Examiner 1 was unaware of the subject’s identities, the examiner who acquired the images, or the specific trial number.

### Statistical analysis

The normality of the data was assessed using the Shapiro-Wilk test. Paired *t* tests were performed to compare medial knee joint space width between the unloaded and loaded conditions.

To assess the intra- and inter-rater reliability, ICCs and 95% confidence intervals were calculated. All ICCs were based on a two-way mixed-effects model for absolute agreement and single-measurement (ICC(3,1)). Intra-rater reliability on the same measurement day was assessed by evaluating the agreement among three repeated measurements performed by each examiner. Intra-rater reliability across separate measurement days was assessed by comparing the first measurement obtained by Examiner 1 on each of the two measurement days. Inter-rater reliability was assessed by comparing first measurements obtained by Examiner 1 on Day 1 and Examiner 2 on Day 2. ICC values were interpreted as follows: greater than 0.90 as excellent, 0.75–0.90 as good, 0.50–0.75 as moderate, and less than 0.50 as poor [21].

Absolute reliability was evaluated using Bland-Altman analysis, which was applied to changes in the medial knee joint space width. Specifically, the analysis included comparisons of measurements taken by Examiner 1 on Day 1 and Day 2 (for intra-rater reliability), as well as by Examiner 1 on Day 1 and Examiner 2 on Day 2 (for inter-rater reliability). Bland-Altman plots were created with the differences between measurements plotted on the *y*-axis and their means on the *x*-axis. Proportional and fixed bias were assessed. If systematic error was detected, the acceptable range of error was calculated. Conversely, in the absence of systematic error, the standard error of measurement (SEM) and the MDC_95_ values were calculated to quantify measurement error.

All statistical analyses were performed using R software (Version 4.6.1; R Foundation for Statistical Computing, Vienna, Austria). The significance level was set at *p* < 0.05.

## Results

The participants had a mean age of 20.2 ± 1.0 years, mean height of 171.2 ± 4.2 cm, and mean weight of 64.0 ± 6.4 kg.

Medial knee joint space width significantly increased under the valgus-loaded condition compared with the unloaded condition (Table 1). ICC(3,1) values for intra-rater reliability of changes in the medial knee joint space width ranged from 0.85 to 0.93 within the same measurement day (Table 2). Across two separate measurement days, the ICC(3,1) value was 0.88 (Table 3). All lower limits of the 95% confidence intervals remained within the good reliability range. The ICC(3,1) value for inter-rater reliability was 0.84; however, the lower limit of its 95% confidence interval fell within the moderate reliability range.

**Table 1:** Comparison of medial knee joint space width under unloaded and loaded conditions (n = 34).

Examiner	Unloaded (mm)	Loaded (mm)	*p* value	Effect size (*d*)
Examiner 1 on Day 1	7.13 ± 1.23	8.46 ± 1.41	< 0.001*	2.55
Examiner 1 on Day 2	7.10 ± 1.11	8.46 ± 1.25	< 0.001*	2.99
Examiner 2 on Day 2	7.16 ± 1.34	8.57 ± 1.48	< 0.001*	2.49

Values are presented as means ± standard deviation.

* *p* < 0.001.

**Table 2:** Intra-rater reliability for the changes in medial knee joint space width within the same measurement day (n = 34).

Examiner	Changes in medial knee joint space width (mm)	ICC(3,1) [95% CI]
Examiner 1 on Day 1	1.33 ± 0.52	0.93 [0.88, 0.96]
Examiner 1 on Day 2	1.36 ± 0.46	0.85 [0.76, 0.92]
Examiner 2 on Day 2	1.41 ± 0.57	0.91 [0.85, 0.95]

Values are presented as means ± standard deviation. ICC(3,1), intraclass correlation coefficient calculated using a two-way mixed-effects model for absolute agreement and single measurements; CI, confidence interval.

**Table 3:** Relative and absolute reliability for changes in medial knee joint space width across two separate measurement days and between examiners (n = 34).

Reliability type	ICC(3,1) [95% CI]	SEM (mm)	MDC_95_ (mm)
Intra-rater	0.88 [0.77, 0.94]	0.19	0.51
Inter-rater	0.84 [0.70, 0.92]	0.23	0.65

ICC, intraclass correlation coefficient; CI, confidence interval; SEM, standard error of measurement; MDC_95_, minimal detectable change at the 95% confidence level.

Intra-rater and inter-rater ICCs were calculated using a two-way mixed-effects model for absolute agreement and single measurements (ICC(3,1)).

**Fig 3:**
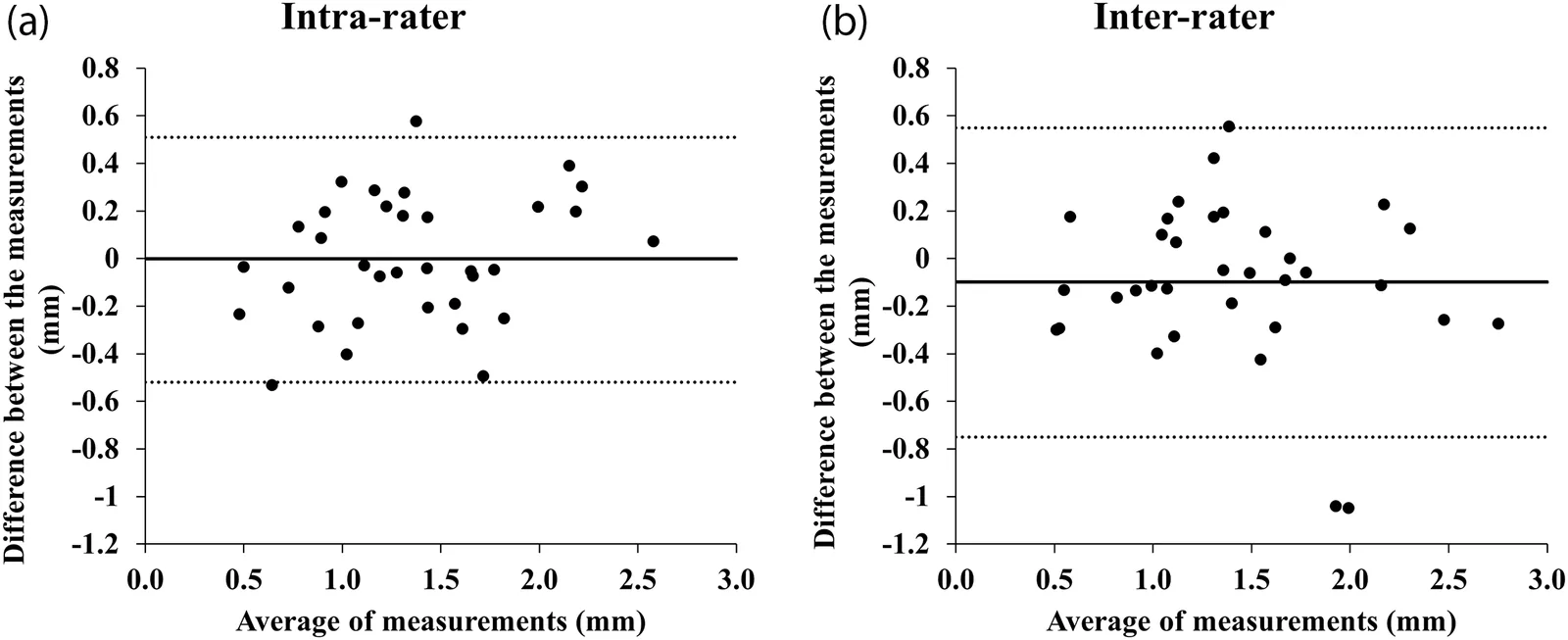
Bland-Altman plots of changes in the medial knee joint space width for (a) intra-rater and (b) inter-rater measurements. The solid line represents the mean difference, and the dotted lines represent the 95% limits of agreement (LoA), calculated as the mean difference ± 1.96 standard deviations.

Figs 3a and 3b show the Bland-Altman plots for intra- and inter-rater measurements. For intra-rater measurements, the 95% limits of agreement ranged from −0.52 to 0.51 mm, with no fixed or proportional bias. The SEM was 0.19 mm, resulting in an MDC_95_ of 0.51 mm. For inter-rater measurements, the 95% limits of agreement ranged from −0.75 to 0.55 mm, with no fixed or proportional bias. The SEM was 0.23 mm, resulting in an MDC_95_ of 0.65 mm. These results indicate that only random errors were observed, demonstrating acceptable absolute reliability for both intra- and inter-rater measurements.

## Discussion

As hypothesized, the measurements exhibited acceptable reliability both within and between examiners. Moreover, the observed measurement errors were primarily random, with the magnitude of errors being less than 1.0 mm for both intra- and inter-rater assessments.

The present study observed a significant increase in the medial joint space width under loaded conditions, accompanied by a large effect size. Slane et al. [10] investigated changes in the medial knee joint space width during valgus stress testing using fresh-frozen cadaveric specimens and found a significant increase in the medial joint space width under loaded conditions compared with unloaded conditions. In contrast, Lutz et al. [11] reported no significant difference in the medial joint space width between unloaded and loaded conditions in healthy individuals. These discrepancies between studies are likely attributed to differences in the methods of applying valgus stress and the amount of load applied to the knee joint. In this study, following the approach used by Slane et al. [10], a lateral force was applied to the distal tibia to induce a valgus stress of 10 Nm on the knee joint. On the other hand, Lutz et al. [11] used a TELOS device to apply 150 N of load to the lateral compartment of the knee joint. Therefore, it is suggested that variations in the magnitude of the applied valgus stress contributed to the differences in the results. Consistent with Slane et al. [10], our findings further support the notion that the valgus stress test leads to an increase in the medial knee joint space width.

The results of this study demonstrated acceptable intra- and inter-rater reliability for ultrasound measurements of changes in medial knee joint space width during valgus stress testing. The ICC point estimates were within the good reliability range, whereas the lower limits of the 95% confidence intervals were 0.77 for intra-rater reliability and 0.70 for inter-rater reliability. These findings suggest that ultrasound measurements can be obtained with acceptable reliability both within and between examiners. Consistent with previous reports [10,11], the present findings support the robustness of ultrasound-based measurements of changes in medial knee joint space width under valgus stress.

In addition to relative reliability, we examined absolute reliability to gain further insight into the precision of the measurement. No fixed or proportional bias was detected, suggesting that the observed disagreement was predominantly random. Averaging measurements across multiple trials is recommended to minimize random error and enhance measurement precision.

The SEM was 0.19 mm for intra-rater measurements and 0.23 mm for inter-rater measurements. The SEM represents the typical magnitude of random measurement error under each reliability condition. Thus, under the present measurement conditions, the typical measurement error was approximately 0.19 mm when measurements were repeated by the same examiner and 0.23 mm for measurements obtained by the two different examiners. The slightly smaller SEM for intra-rater measurements suggests that measurements performed by the same examiner may provide greater precision than those performed by different examiners.

The MDC_95_, which represents the minimum amount of change required to exceed measurement error with 95% confidence, was 0.51 mm for intra-rater measurements and 0.65 mm for inter-rater measurements. These values provide practical thresholds for determining whether an observed change in medial knee joint space width during valgus stress testing exceeds measurement error under the present measurement conditions. When measurements are repeated by the same examiner, a change greater than 0.51 mm would be likely to represent a change beyond measurement error, whereas a change greater than 0.65 mm would be required for measurements obtained by the two different examiners included in this study. Conversely, changes smaller than these thresholds should be interpreted cautiously because they cannot be confidently distinguished from measurement error. These findings also suggest that using the same examiner may be preferable when monitoring small changes over time.

Both the SEM and MDC_95_ were below 1.0 mm. Given that the mean valgus stress-induced changes in medial knee joint space width were approximately 1.3–1.4 mm, these values were small relative to the magnitude of the observed widening. Together with the significant differences between unloaded and loaded conditions, these findings suggest that ultrasound imaging may provide sufficient precision to evaluate valgus stress-induced changes in medial joint space width in healthy knees.

The International Knee Documentation Committee 2000 (IKDC 2000) Objective Examination Form [22] classifies a difference of 0–2 mm in medial joint opening between the involved knee and the normal or presumed-normal knee as normal. However, direct comparison of this criterion with the present findings is difficult because the IKDC classification is based on side-to-side differences, whereas the present study quantified changes between unloaded and valgus-loaded conditions within the same knee. Nevertheless, the SEM and MDC_95_ values obtained in the present study were less than 1.0 mm, indicating that both the measurement error and detectable-change threshold were small relative to the millimeter-scale differences used in the IKDC 2000 classification of knee ligament laxity. This level of measurement precision suggests the potential clinical utility of ultrasound imaging for quantifying medial knee laxity, although its applicability to pathological knees remains to be established.

This study has several limitations that should be addressed. First, the sample size was originally determined to detect significant changes in medial knee joint space width rather than to evaluate reliability. A post hoc assessment based on the method proposed by Walter et al. [23] suggested that a larger sample size than that used in the present study would have been desirable for reliability studies. Therefore, the reliability estimates obtained in this study should be interpreted with some caution. Nevertheless, the 95% confidence intervals for the ICC estimates were reasonably narrow, suggesting that the reliability estimates were obtained with acceptable precision. Future studies with larger sample sizes are warranted to further confirm the reliability of ultrasound measurements of changes in medial joint space width during valgus stress testing. Second, the participants were individuals with healthy knees, and it remains unclear whether the acceptable reliability of both intra- and inter-rater assessments observed in this study would apply to other populations, particularly those with knee injuries. Additionally, it is uncertain whether the ability of ultrasound imaging to quantify medial knee joint laxity is comparable to that of stress radiography. Therefore, future research should focus on assessing changes in the medial knee joint space width during valgus stress testing in individuals with medial knee joint laxity. Third, this study included only male participants to minimize confounding factors associated with hormonal fluctuations that can affect joint laxity. However, while MCL injuries have been reported to occur more frequently in males than in females [3,4], they remain a common injury in females. Consequently, the exclusion of female participants limits the generalizability of our findings. Further studies involving female participants are essential to ensure broader clinical applicability. Fourth, valgus stress-induced changes in medial knee joint space width may depend on the method used to apply valgus stress and the magnitude of the applied load. In this study, a licensed physical therapist with over 10 years of experience in musculoskeletal rehabilitation manually applied the valgus stress to the knee. Although the use of a handheld dynamometer offers significant advantages in terms of clinical utility and portability, and the examiner’s expertise contributes to consistency, the reliability of the results may still be influenced by individual skill levels, unlike fixed mechanical devices. Finally, regarding the applied valgus load, the force was standardized at 10 Nm, as this level was confirmed to be safe in previous studies [8,10,19]. This decision was informed by previous findings from LaPrade et al. [8], who reported that forces of 15 Nm and 20 Nm caused damage to the medial structures of the knee in cadaveric specimens. However, the magnitude of the applied load must be set with great caution when performing a valgus stress test in patients with MCL injuries or immediately after reconstruction surgery, because the valgus stress may impose tensile loads on the ligaments, which carries a high risk of inducing pain. As a result, it may not be possible to apply sufficient valgus stress in clinical settings involving injured or postoperative patients, potentially affecting the degree of medial joint space widening and compromising the reliability of the measurements. Therefore, it is necessary to investigate whether measurements can be reliably obtained under similar loading conditions and environments in actual patients with ligament injuries or immediately after reconstruction surgery. Clarifying these aspects would contribute to the clinical applicability of medial joint space evaluation using ultrasound imaging, both for assessing ligament laxity and for postoperative monitoring following ligament reconstruction.

## Conclusions

This study evaluated both the relative and absolute reliability of ultrasound-based measurements of changes in medial knee joint space width under valgus stress in healthy individuals. The findings demonstrated acceptable intra- and inter-rater reliability, with measurement errors being predominantly random and consistently less than 1.0 mm. These findings suggest that ultrasound imaging could provide sufficient reliability to quantify changes in medial joint space width. However, to establish its clinical utility in assessing knee joint laxity, further investigations involving individuals with ligamentous knee injuries are warranted.

## Supporting information

S1 Data Subject characteristics and ultrasound measurement data. The Excel file contains four worksheets: subject characteristics (Sheet 1), ultrasound measurement data obtained by Examiner 1 on Day 1 (Sheet 2), ultrasound measurement data obtained by Examiner 1 on Day 2 (Sheet 3), and ultrasound measurement data obtained by Examiner 2 on Day 2 (Sheet 4)
(XLSX).
